# Left innominate vein stenosis in an asymptomatic population: a retrospective analysis of 212 cases

**DOI:** 10.1186/s40001-017-0243-3

**Published:** 2017-01-23

**Authors:** Xiangjiang Guo, Yaxue Shi, Hui Xie, Lan Zhang, Guanhua Xue, Leyi Gu, Changning Hao, Shuofei Yang, Kejia Kan

**Affiliations:** 1 Department of Vascular Surgery, Renji Hospital, School of Medicine, Shanghai Jiao Tong University, Shanghai, China; 2 Department of Vascular Surgery, LONGHUA Hospital, Shanghai University of Traditional Chinese Medicine, No.725 Wanping South Road, Shanghai, 200032 China; 3 Department of Nephrology, Renji Hospital, School of Medicine, Shanghai Jiao Tong University, Shanghai, China

**Keywords:** Left innominate vein stenosis, Asymptomatic patients, MDCT angiography, Risk factors

## Abstract

**Background:**

Although left innominate vein (LIV) stenosis has been demonstrated to be attributed to compression by adjacent anatomical structures, most of the studies are focusing on hemodialysis patients with clinical symptoms compatible with LIV stenosis. The goal of this study was to retrospectively investigate the incidence of LIV stenosis and its influencing factors in an asymptomatic, non-hemodialysis population, which has rarely been performed.

**Methods:**

From Jan 2013 to Dec 2014, 212 consecutive cases undergoing a chest multi-detector computed tomography (MDCT) angiography were enrolled. LIV stenosis was defined as loss of the area of the LIV (that is, 1 − compression degree) >25%. Multivariate logistic regression analysis was performed to explore the independent risk factors associated with LIV stenosis.

**Results:**

LIV stenosis occurred in 35.4% of cases (75/212), with the median loss of the area of the LIV of 36.2% (interquartile range 30.2–49.8%). There were significant differences in age (62.5 ± 11.7 vs. 58.6 ± 14.3 years; *P* = 0.041), BMI (23.9 ± 2.9 vs. 23.0 ± 3.3, *P* = 0.036), the frequency of crossing site of LIV over the origin of the aortic arch (54.7 vs. 24.8%, *P* < 0.001), and the space between aortic arch and sternum [mean ± SD, 11.6 ± 4.2 mm vs. median, 14.1 (interquartile range 11.9–16.3) mm, *P* < 0.001] between patients with and without LIV stenosis, but only the latter two were confirmed as independent factors by the multivariate logistic regression analysis [crossing site of LIV over the aortic arch, OR (95% CI) = 2.632 (1.401, 4.944), *P* = 0.003; space between the aortic arch and sternum, OR (95% CI) = 0.841 (0.770, 0.919), *P* < 0.001].

**Conclusion:**

The patients with an older age, high BMI, LIV crossing over the origin of the aortic arch, or smaller space between aortic arch and sternum may have high risks for LIV stenosis. They should be paid more attention to exclude LIV stenosis preoperatively using MDCT angiography to prevent venous access dysfunction and symptomatic development by fistula creation when hemodialysis is required.

## Background

Left innominate vein (LIV) stenosis is previously considered to be a common and serious complication due to previous venous catheterization [1, 2], pacemakers, or defibrillators implantation [3, 4]. Although recent studies indicate extrinsic compression of LIV by adjacent anatomical structures, such as the aortic arch, innominate artery and sternum may be another mechanism for LIV stenosis [5–7]. However, most of these studies are focusing on hemodialysis patients with clinical symptoms compatible with LIV stenosis, including arm edema, arm ulceration, ipsilateral face swelling, visible collateral veins, and venous dilatation [7–9]. For example, KOTODA et al. reported that LIV stenosis occurred in seven hemodialysis patients using digital subtraction angiography (DSA) and confirmed an anatomic relationship among the LIV, sternum, and arch vessels by multi-detector computed tomography (MDCT) angiography [9]. Further, Shi et al. [8] proved that these anatomical factors to contribute to LIV stenosis in hemodialysis patients by measuring the LIV diameter as well as the space between the sternum and aortic arch on the cross section of the MDCT scan. Rare studies to investigate the anatomical compression mechanism of LIV stenosis in non-hemodialysis patients without corresponding clinical symptoms. Therefore, the goal of this study was to retrospectively investigate the incidence of LIV stenosis and its influencing factors in an asymptomatic, non-hemodialysis population using MDCT angiography.

## Methods

### Patients

From Jan 2013 to Dec 2014, 212 consecutive outpatients who underwent chest MDCT angiography examination in Renji Hospital, School of Medicine, Shanghai JiaoTong University due to other primary diseases (such as chest pain, heart disease, lung disease, etc.), were enrolled in this study. Patients were excluded from analysis if they met any of the exclusion criteria: (1) aged less than 20 years, (2) with a huge occupying lesion in neck or thoracic cavity, (3) had thoracocyllosis, (4) gravida, (5) had aneurysm of thoracic aorta, (6) had a history of venous placement of catheters, pacemaker, and defibrillator wires, or (7) had clinical signs of LIV compression syndrome. This study was approved by the Ethics Committee of Renji Hospital.

### Data collection

Medical records of the eligible patients including demographic data, current diseases, and history of smoking and thoracic surgery were collected. All patients underwent an MDCT scan examination using a BrightSpeed Elite CT scanner with a spatial resolution of 0.625 mm (GE, Milwaukee, USA) in a supine position. Scanning parameters were (1) axial images, 1.25–5 mm with an interval of 1–5 mm; (2) rotation speed, 0.35 s; and (3) table speed, 7.5–15 mm/s. Images were acquired during maximum inspiration and breath holding. Non-ionic contrast medium (1.8 ml/kg body weight; iopamidol 370, Bracco Sine Pharmaceutical Co. Ltd., Shanghai, China) was administered using a power injector through the right arm vein at a speed of 3–3.5 ml/s. The scan images were reviewed by two investigators using GE AW VOLUMESHARE2 Workstations. The space between the aortic arch and sternum, diameter of the aortic arch, anterior–posterior diameter and left–right diameter of the thoracic cage, and length of anterior and posterior mediastinum were measured. Also, the areas of LIV at its crossing site over aortic arch, as well as areas of LIV proximal and distal to the crossing site, were also recorded. The degree of compression was expressed as the ratio of the area of the LIV at the site of maximal compression (that is, residual surface area) to area of the un-compressed distal ends of LIV. LIV stenosis was defined as loss of the area of the LIV (that is, 1 − compression degree) >25%.

### Statistical analysis

Data were analyzed using SPSS 19.0 software (SPSS Inc., Chicago, IL, USA). Normally and non-normally distributed continuous data were expressed as the mean ± standard deviation (SD) and median (interquartile range, IQR), respectively. Differences of continuous data were compared using independent *t* test or Mann–Whitney *U* test when appropriate. Categorical data were shown as frequency (proportion) and compared using *χ* ^2^ test. Only significant variables with a *P* value <0.05 on the univariate analysis were entered into the multivariate logistic regression model with a forward feature selection procedure to investigate the independent risk factors associated with LIV stenosis.

## Results

**Fig. 1:**
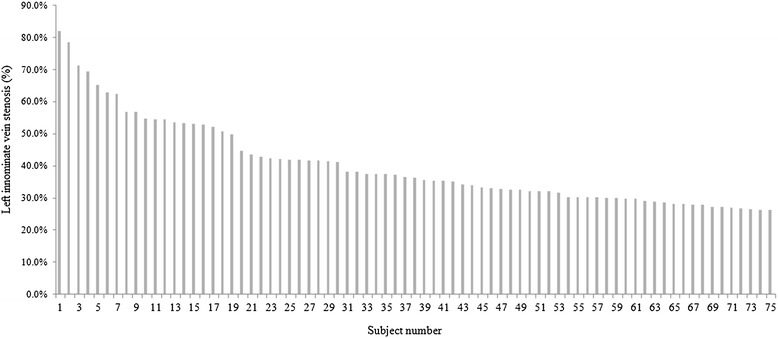
The distribution of left innominate vein stenosis in each subject

A total of 212 asymptomatic subjects including 122 males and 90 females (mean age 60.0 ± 13.5 years) were retrospectively reviewed. MDCT scan revealed that LIV stenosis occurred in 35.4% of cases (75/212), with the median loss of the area of the LIV of 36.2% (IQR, 30.2–49.8%) (Fig. 1).

Table 1 shows the comparison of demographic and anatomical characteristics between patients with and without LIV stenosis. As a result, there were no significant differences in demographic characteristics of gender (*P* = 0.134), height (*P* = 0.059), weight (*P* = 0.372), hypertension (*P* = 0.059), diabetes mellitus (*P* = 0.968), coronary disease (*P* = 0.400), stroke (*P* = 0.388), history of smoking (*P* = 0.663), thoracic surgery (*P* = 0.345), and calcification of aortic arch (*P* = 0.557) between two groups; however, LIV stenosis patients showed an older age (62.5 ± 11.7 vs. 58.6 ± 14.3 years, *P* = 0.041) and a higher BMI (23.9 ± 2.9 vs. 23.0 ± 3.3, *P* = 0.036).

**Table 1:** Comparison of characteristics in patients with and without LIV stenosis

Risk factors	LIV stenosis		*P*
	Without (*n* = 137)	With (*n* = 75)	
Male, *n* (%)	84 (61.3)	38 (50.7)	0.134
Age, years	58.6 ± 14.3	62.5 ± 11.7	0.041
Height, cm	166.3 ± 7.6	164.2 ± 8.0	0.059
Weight, kg	62 (56–70)	65 (60–70)	0.372
BMI	23.0 ± 3.3	23.9 ± 2.9	0.036
Hypertension, *n* (%)	31 (22.6)	26 (34.7)	0.059
Diabetes mellitus, *n* (%)	18 (13.1)	10 (13.3)	0.968
Coronary disease, *n* (%)	10 (7.3)	8 (10.7)	0.400
Stroke, *n* (%)	4 (2.9)	4 (5.3)	0.388
History of smoking, *n* (%)	25 (18.4)	12 (16.0)	0.663
History of thoracic surgery, *n* (%)	21 (15.3)	8 (10.7)	0.345
Calcification of aortic arch, *n* (%)	51 (37.2)	31 (41.3)	0.557
Crossing site of LIV over aortic arch, *n* (%)			<0.001
Over the origin of the aortic arch	34 (24.8)	41 (54.7)	
Over the three branches of the aortic arch	103 (75.2)	34 (45.3)	
Space between the aortic arch and sternum, mm	14.1 (11.9–16.3)	11.6 ± 4.2	<0.001
Aortic arch diameter, mm	30.2 ± 4.3	30.8 ± 3.8	0.262
Aortic arch cross-sectional area, mm^2^	728.9 ± 205.7	757.6 ± 177.2	0.310
Anterior–posterior diameter of the thoracic cage, mm	165.7 ± 19.9	168.3 ± 23.9	0.408
Left–right diameter of the thoracic cage, mm	228.5 ± 36.6	219.2 ± 41.4	0.094
Length of anterior and posterior mediastinum, mm	81.0 (71.4–95.2)	80.8 (69.9–100.4)	0.822

*LIV* left innominate vein, *BMI* body mass index

As to the anatomical factors (Table 1), there were no statistical differences between patients with and without LIV stenosis in the aortic arch diameter (30.2 ± 4.3 vs. 30.8 ± 3.8 mm, *P* = 0.262), aortic arch cross-sectional area (728.9 ± 205.7 vs. 757.6 ± 177.2 mm^2^, *P* = 0.310), anterior–posterior diameter of the thoracic cage (165.7 ± 19.9 vs. 168.3 ± 23.9 mm, *P* = 0.408), left–right diameter of the thoracic cage (228.5 ± 36.6 vs. 219.2 ± 41.4 mm, *P* = 0.094), and length of anterior and posterior mediastinum [81.0 (71.4–95.2) vs. 80.8 (69.9–100.4) mm, *P* = 0.822]. Nevertheless, more patients having the LIV crossed over the origin of the aortic arch in the LIV stenosis group [54.7% (41/75) vs. 24.8% (34/137), *P* < 0.001]. Also, the space between the aortic arch and sternum was significantly smaller in the patients with LIV stenosis than that in patients without LIV stenosis [mean ± SD, 11.6 ± 4.2 mm vs. median, 14.1 (IQR 11.9–16.3) mm, *P* < 0.001].

Multivariate logistic regression analysis showed that only the crossing site of LIV over the aortic arch [OR (95% CI) = 2.632 (1.401, 4.944), *P* = 0.003] and space between the aortic arch and sternum were independent factors for LIV stenosis [OR (95% CI) = 0.841 (0.770, 0.919), *P* < 0.001] (Table 2). These findings suggested that patients with LIV crossing over the origin of the aortic arch or patients with smaller space between aortic arch and sternum had higher risks for LIV stenosis.

**Table 2:** Identification of independent risk factors for LIV stenosis

Risk factors	Multivariable logistic regression analysis		
	β	OR (95% CI)	*P*
BMI	0.092	1.097 (0.990–1.215)	0.078
Crossing site of LIV over the aortic arch^a^	0.968	2.632 (1.401, 4.944)	0.003
Space between the aortic arch and sternum	−0.173	0.841 (0.770, 0.919)	<0.001

*LIV* left innominate vein, *OR* odd ratio, *CI* confidence interval, *BMI* body mass index

^a^The category “over the three branches of aortic arch” was used as the reference category

## Discussion

Most researches focus on LIV compression, stenosis, or occlusion in hemodialysis patients [7–9]. To the best of our knowledge, this is the first report of LIV stenosis in non-hemodialysis, asymptomatic patients with MDCT angiography images. Our results demonstrated a prevalence of 35.4% in the investigated population with the median loss of the area of the LIV of 36.2% (IQR 30.2–49.8%), which seemed to be relatively lower than that in the hemodialysis, symptomatic patients (44%, 21/48 [5]; and 47.4%, 9/19 [8]). This may be attributed to the fact that in non-hemodialysis patients, LIV compression can be partially compensated by collateral veins along the chest wall, in the neck, and in the mediastinum, thus avoiding the development of LIV stenosis and corresponding symptoms [5, 8]. On the contrary, creation of arteriovenous fistula for hemodialysis patients may lead to remarkably increased blood flow through the central veins, which is not insufficient to be compensated by collateral veins, thus causing venous hypertension and related clinical symptoms [5, 8]. Accordingly, our study indicates the necessity of preoperative evaluation of LIV stenosis, aiming to prevent the development of clinical symptoms by fistula creation when hemodialysis is required.

It is believed that tortuosity and/or expansion of the great vessels may result in the above extrinsic compression [5], while the aortic or innominate artery may become tortuous and ectatic with aging, systemic hypertension, and high BMI [10, 11]. Therefore, these demographic characteristics may serve as risk factors for LIV stenosis theoretically. As expected, age and BMI were found to be significantly different between patients with and without LIV stenosis. The insignificant difference in hypertension may be due to the small sample size in our study.

Furthermore, our multivariate logistic regression analysis showed that only the crossing of LIV over the origin of the aortic arch and small space between aortic arch and sternum were independent factors for LIV stenosis, further confirming the fact that LIV stenosis may be caused by anatomical compression of the aortic arch behind the sternum, which was in line with previous studies [6, 8].

Digital subtraction angiography (DSA), which can clearly visualize the blood vessels, is the gold standard for detecting angiostenosis [12, 13]. However, its utilization is limited by failing to identify extrinsic compression. In addition to assessing the vascular access condition and degree of vascular stenosis, MDCT angiography is able to rebuild the images of surrounding structures around the vascular stenosis site [14–17]. Hence, the patients who underwent the MDCT angiography examination were included in our analyses. Furthermore, it is reported that the contrast media are injected by the right arm veins during the MDCT scan which may provide better image quality compared with the left arm injection [18]. Thus, we also adopted the right arm injection in our study.

Our study had some limitations. First, the retrospective nature may result in some data recording bias. Second, the number of patients included in this study was small, which may lead to an under- or over-estimation of LIV stenosis and the roles of its influencing factors. Thirdly, our patients were collected from outpatient clinics, and most of them were not subjected to vascular diseases. Thus, DSA examination was not performed and the comparison between DSA and MDCT could not be available. Accordingly, we believe our conclusions should be further evaluated and confirmed in greater detail in a larger prospective study.

## Conclusion

Our present study indicates that there may also be approximately 35.4% patients to have LIV stenosis in an asymptomatic, non-hemodialysis population. The patients with an older age, high BMI, LIV crossing over the origin of the aortic arch, or smaller space between aortic arch and sternum may have high risks for LIV stenosis. Therefore, these patients should be paid more attention to exclude LIV stenosis preoperatively using MDCT angiography to prevent venous access dysfunction and symptomatic development by fistula creation when hemodialysis is required.

## Abbreviations

LIV: left innominate vein
MDCT: multi-detector computed tomography
SD: standard deviation
IQR: interquartile range
DSA: digital subtraction angiography
